# Gut microbiota composition and *Clostridium difficile* infection in hospitalized elderly individuals: a metagenomic study

**DOI:** 10.1038/srep25945

**Published:** 2016-05-11

**Authors:** Christian Milani, Andrea Ticinesi, Jacoline Gerritsen, Antonio Nouvenne, Gabriele Andrea Lugli, Leonardo Mancabelli, Francesca Turroni, Sabrina Duranti, Marta Mangifesta, Alice Viappiani, Chiara Ferrario, Marcello Maggio, Fulvio Lauretani, Willem De Vos, Douwe van Sinderen, Tiziana Meschi, Marco Ventura

**Affiliations:** 1 Laboratory of Probiogenomics, Department of Life Sciences, University of Parma, Italy; 2 Internal Medicine and Critical Subacute Care Unit, Parma University Hospital, Parma, Italy; 3 Department of Clinical and Experimental Medicine, University of Parma, Parma, Italy; 4 Laboratory of Microbiology, Wageningen University, Dreijenplein 10, 6703 HB, Wageningen, The Netherlands; 5 GenProbio srl, Parma, Italy; 6 Geriatric Unit, Parma University Hospital, Parma, Italy; 7 APC Microbiome Institute and School of Microbiology, Bioscience Institute, National University of Ireland, Cork, Ireland

## Abstract

The gut microbiota composition of elderly hospitalized patients with *Clostridium difficile* infection (CDI) exposed to previous antibiotic treatment is still poorly investigated. The aim of this study was to compare the microbiota composition by means of 16S rRNA microbial profiling among three groups of hospitalized elderly patients (age ≥ 65) under standard diet including 25 CDI-positive (CDI group), 29 CDI-negative exposed to antibiotic treatment (AB+ group) and 30 CDI-negative subjects not on antibiotic treatment (AB− group). The functional properties of the gut microbiomes of CDI-positive vs CDI-negative subjects were also assessed by shotgun metagenomics. A significantly lower microbial diversity was detected in CDI samples, whose microbiomes clustered separately from CDI-negative specimens. CDI was associated with a significant under-representation of gut commensals with putative protective functionalities, including *Bacteroides, Alistipes, Lachnospira* and *Barnesiella*, and over-representation of opportunistic pathogens. These findings were confirmed by functional shotgun metagenomics analyses, including an in-depth profiling of the Peptostreptococcaceae family. In CDI-negative patients, antibiotic treatment was associated with significant depletion of few commensals like *Alistipes*, but not with a reduction in species richness. A better understanding of the correlations between CDI and the microbiota in high-risk elderly subjects may contribute to identify therapeutic targets for CDI.

The incidence of *Clostridium difficile* (recently classified as *Peptoclostridium difficile*) infections^1^ (CDI) has dramatically risen in industrialized countries in the last fifteen years^2^. Although community-based cases are more and more common, CDI mainly affects hospitalized patients, especially multimorbid elderly^3^,^4^,^5^. Current therapeutic strategies, including oral vancomycin and metronidazole, have limited efficacy and treatment failure may occur in as much as one third of cases^6^.

Antibiotic treatment is considered the most important risk factor for CDI^7^,^8^. In fact, it may result in gut microbiota alterations, whose onset may be crucial for the loss of colonization resistance to *C. difficile*^9^,^10^. Some studies carried out in both mouse models and humans have shown that different types of antibiotic treatment can affect gut microbiota composition in different ways, resulting in different levels of CDI risk^11^,^12^. Adverse alterations in gut microbiota composition are considered to play a central role in the development, maintenance as well as recurrence of CDI, and the recent therapeutic successes of fecal microbiota transplantation (FMT) in highly-recurrent or refractory CDI support this notion^13^.

Currently, the precise microbiome alterations associated with CDI have not been completely elucidated in the high-risk population of elderly hospitalized subjects. Previous studies have focused on case-series of adult subjects, mainly employing low-throughput methodological approaches in microbiome analyses^14^,^15^,^16^,^17^,^18^,^19^. So far, no studies have investigated the gut microbiota composition by means of both deep-sequencing 16S rRNA profiling and shotgun metagenomics in elderly multimorbid hospitalized patients stratified for CDI and antibiotic exposure^20^. Interestingly, the ageing process has recently been associated with alterations in gut microbiota composition, being strongly influenced by diet, institutionalization and age itself, with the highest level of gut microbiota alterations detected in centenarians^21^,^22^.

In this cross-sectional study, by using 16S rRNA microbial profiling and shotgun metagenomics, we aimed to determine and compare the composition of fecal microbiota among three groups of multimorbid hospitalized elderly, categorized according to the presence of CDI and exposure to antibiotic treatment.

## Materials and Methods

### Study population

At the Internal Medicine and Critical Subacute Care Unit of Parma University Hospital, Italy, we enrolled three groups of elderly (≥65 years old) multimorbid (≥2 chronic diseases) patients acutely hospitalized for extra-intestinal illness from November 2014 to April 2015. The case-mix of patients admitted to our unit and the clinical setting have been recently described in a case study^23^ and include a wide range of acute diseases such as pneumonia, urinary tract infections, respiratory insufficiency, acute pulmonary edema, syncope and delirium. Subjects with inflammatory bowel disease (IBD), coeliac disease, intestinal cancer, pancreatic or liver insufficiency, dysphagia, active artificial nutrition (either enteral or parenteral) and terminal disease (established prognosis quoad vitam <30 days) were excluded from the study. All participants at the time of enrolment followed the same hospital-based oral diet, characterized by a balanced intake of macronutrients and an energy intake of 1700 Kcal/day.

*Clostridium difficile*-infected (CDI) group was composed of 25 subjects diagnosed with hospital-acquired active CDI, irrespective of previous antibiotic treatment, enrolled before initiation of metronidazole or oral vancomycin. According to guidelines^24^, CDI was diagnosed when both *C. difficile* glutamate dehydrogenase (GDH) enzyme immunoassay and Polymerase-Chain Reaction (PCR) were positive in at least one stool sample from a patient with diarrhea or visualization of pseudomembranes on colonoscopic examination. Diarrhea was defined as three or more loose bowel movements per day, with no other known cause.

Antibiotic-exposed (AB+) group was composed of 29 subjects without diarrhea or other symptoms of CDI, admitted for extra-intestinal infectious disease (pneumonia, urinary-tract infection or soft tissue infection) and exposed to antibiotic treatment during their hospital stay.

Non-antibiotic exposed group (AB−) group included 30 patients without diarrhea or other symptoms of CDI, admitted for extra-intestinal non-infectious disease (including cardiovascular disease, respiratory failure or neurological disease) and not exposed to antibiotic treatment during their hospital stay.

A fecal sample of at least 2 grams was obtained for every patient, immediately frozen at −20 °C and subsequently delivered to Laboratory of Probiogenomics of Parma University for processing and analysis. Fecal samples of subjects with suspected CDI were collected at the time of onset of symptoms. Those not confirmed as CDI-positive by microbiological analysis or colonoscopic examination were not considered for final analysis.

The study protocol was approved by the Ethics Committee of the University of Parma (ID 14091). Informed consent was obtained from all participants. All investigations were carried out following the principles of the Declaration of Helsinki.

### Clinical data collection

For each participant, data about main diagnosis, comorbidities, number and type of chronic medications, frailty, weight and duration of hospital stay were recorded. Namely, the overall burden of multimorbidity was calculated through Cumulative Illness Rating Scale (CIRS) Severity index^25^. Frailty was assessed according to cumulative deficit model (Rockwood Clinical Frailty Scale)^26^. Exposure to proton pump inhibitors (PPIs) and antibiotics was particularly assessed, given their possible interaction with gut microbiota.

### 16S rRNA gene amplification

Partial 16S rRNA gene sequences were amplified from the bacterial DNA extracted from stool samples using primer pair Probio Uni and/Probio_Rev, which targets the V3 region of the 16S rRNA gene sequence^27^. Illumina adapter overhang nucleotide sequences were added to the 16S rRNA gene-specific sequences. The 16S rRNA gene amplicons were prepared following the 16S Metagenomic Sequencing Library Preparation Protocol. Amplifications were carried out using a Verity Thermocycler (Applied Biosystems). The integrity of the PCR amplicons was analysed by electrophoresis on a 2200 TapeStation Instrument (Agilent Technologies, USA).

### MiSeq sequencing of 16S rRNA Gene-based amplicons

PCR products obtained after amplification of the 16S rRNA gene sequences were purified by magnetic purification step involving the Agencourt AMPure XP DNA purification beads (Beckman Coulter Genomics GmbH, Bernried, Germany) in order to remove primer dimers. DNA concentration of the amplified sequence library was estimated through fluorimetric Qubit quantification system (Life Technologies). Amplicons were diluted to 4 nM, and 5 μl of each diluted DNA amplicon solution was mixed to prepare the pooled final library. Sequencing was performed using an Illumina MiSeq sequencer with MiSeq Reagent Kit v3 chemicals.

### 16S rRNA-based Microbiota Analysis

The fastq files were processed using QIIME^28^ as previously described^27^. Paired-end reads were merged and quality control retained sequences with a length between 140 and 400 bp, mean sequence quality score >25 and with truncation of a sequence at the first base if a low quality rolling 10 bp window was found. Sequences with mismatched forward and/or reverse primers were omitted.

### Shotgun metagenomics

This additional technique was performed on 15 samples (5 CDI, 5 AB+, 5 AB−) randomly selected from the whole population. Bacterial DNA was extracted from fecal samples using the QIAamp DNA Stool Mini kit following the manufacturer’s instructions (Qiagen Ltd., Strasse, Germany) and subsequently fragmented to 550–650 bp using a BioRuptor machine (Diagenodo, Belgium). Samples were prepared following the TruSeq Nano DNA Sample Preparation Guide. Sequencing was performed using an Illumina MiSeq sequencer with MiSeq Reagent Kit v3 chemicals.

### Analysis of metagenomic datasets

The fastq were filtered for reads with quality <25 and presence of alien DNA, as well as for reads <80 bp. Bases were also removed from the end of the reads until the average quality in a window of 5 bp was >25. Only paired data was further analyzed. Taxonomic classification of the reads was obtained using MEGAnnotator software^29^ for homology searches in the NCBI nr database, followed by data processing using MEGAN5 software^30^. Reads screening for antibiotic resistance genes was performed using a custom script based on RapSearch2 software^31^, htseq-count^32^ and the databases CARD^33^ and TCDB^34^ in order to encompass both enzymes and transporters involved in antibiotic resistance. Reconstruction of GH profiles as well as bacterial metabolic pathways and evaluation of their abundance in the shotgun metagenomics datasets was performed using custom scripts based on RapSearch2 software^31^, htseq-count^32^ and the CAZy database^35^ or the MetaCyc database^36^, respectively. Profiling of the Peptostreptococcaceae family was performed by mapping of shotgun metagenomics reads on the genome of Peptostreptococcaceae members public available (listed in Fig. S4) with addition of *Romboutsia* spp. FRIFI through BWA software. Ribosomal loci and mobile elements of these genomes where excluded from the mapping in order to avoid false positives and htseq-count^32^ was employed for reads counts.

### Statistical analyses

Kruskal-Wallis one-way analysis of variance adjusted for age and sex was performed with SAS statistical package, version 9.1 (SAS Institute Inc., Cary, North Carolina) software, in order to compare among the three study groups: a) clinical variables, b) α- and β-diversity indexes of species richness, and c) the average relative abundance of putative bacterial taxa shared among at least two groups and with a minimum 0.1% representation in at least one group. For those taxa with an age- and sex-adjusted statistically significant difference (p < 0.05) among two groups, a fully-adjusted statistical model was also built, accounting for clinical variables that were significantly different among the three groups (i.e. duration of hospital stay and number of drugs).

PERMANOVA and Kendall Tau-rank co-occurrence analyses were also performed, using QIIME^28^ and QSPSS software (www.ibm.com/software/it/analytics/spss/) respectively. Co-occurrence data and Cytoscape^37^ were used to build a force-driven network map representing the effect of the depletion of key taxa in PDI group on the global microbial ecology of fecal samples.

### Availability of supporting data

The 16S rRNA profiling data and shotgun metagenomics data sequenced in this study were deposited in SRA database under the following study accession numbers: PRJNA297268 and PRJNA297269.

## Results

### Clinical features of participants and 16S rRNA profiling output

The study population (84 hospitalized subjects, aged ≥65) was composed of three groups that were similar for age, weight, functional disabilities and multimorbidity burden (Table 1), and followed the identical hospital-based standard oral diet for at least three days prior sampling of feces. The CDI group was composed of 25 subjects with laboratory-confirmed CDI, enrolled at the time of onset of symptoms, irrespective of previous antibiotic exposure and before the initiation of metronidazole or oral vancomycin treatment. The AB+ group included 29 CDI-negative subjects admitted for extra-intestinal infectious diseases and exposed to antibiotic treatment. The AB− group encompassed 30 CDI-negative patients admitted for extra-intestinal non-infectious diseases and thus not exposed to antibiotic treatment.

Next Generation sequencing followed by quality and chimera filtering of the 84 samples produced a total of 3,903,701 filtered reads with an average of 46,473, and ranging from 11,717 to 104,706 (Table S1). The level of biodiversity coverage was assayed through both Shannon and Chao1 biodiversity curves, which tended to plateau for each sample, confirming the high accuracy of the performed 16S rRNA profiling analysis.

### Comparison of the gut microbiota intra- and inter-individual variability among the three groups

**Figure 1:**
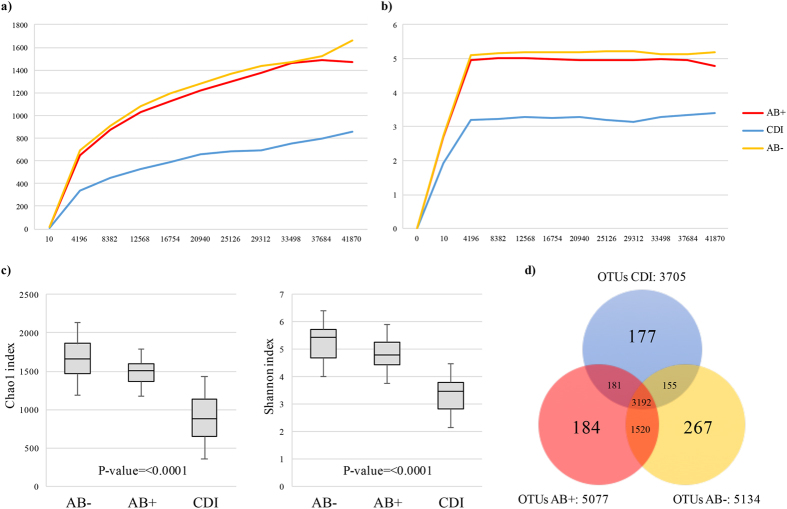
Evaluation of alpha-diversity in AB−, AB+ and CDI samples. Panel (**a**) shows the average rarefaction curves representing variation of the Chao1 diversity index at increasing sequencing depth of AB−, AB+ and CDI samples. Panel (**b**) displays the average rarefaction curves representing variation of the Shannon diversity index at increasing sequencing depth of AB−, AB+ and CDI samples. Panel (**c**) represents a box plot showing standard deviation of the Chao1 and Shannon indexes calculated by subsampling AB−, AB+ and CDI datasets at 41,870 sequences (representing the highest subsampling performed). Panel (**d**) depicts a Venn diagram illustrating the total, unique and shared number of OTUs predicted for AB−, AB+ and CDI datasets.

We evaluated the differences in bacterial population diversity index (alpha diversity) between AB−, AB+ and CDI patients. Shannon and Chao1 biodiversity indexes produced different average rarefaction curves among the three groups (Fig. 1). Fecal samples from AB+ showed a similar level of gut microbiota complexity as compared to AB− (Fig. 1). In contrast, CDI patients displayed a lower level of biodiversity than AB+ (Fig. 1). Such differences were confirmed as statistically significant based on age- and sex-adjusted Kruskal-Wallis one-way analysis of variance (p-value < 0.0001) calculated at the highest available sequencing depth (Fig. 1).

16S rRNA-predicted Operational Taxonomic Units (OTUs) clustered at 97% identity were employed to determine the unique and shared members of the bacterial gut population present in the three groups of samples. The total number of identified OTUs supported by at least 10 amplicons was 5676. Among them, 3192 were shared by all groups, while 5134, 5077 and 3705 represented the total pool associated with AB−, AB+ and CDI subjects, respectively (Fig. 1). Notably, 267, 184 and 177 OTUs were uniquely present in the AB−, AB+ and CDI subjects, respectively. Moreover, we identified 181 OTUs that were shared between AB+ and CDI, while 1520 and 155 OTUs were shared by samples from AB− and AB+, and AB− and CDI subjects, respectively (Fig. 1).

**Figure 2:**
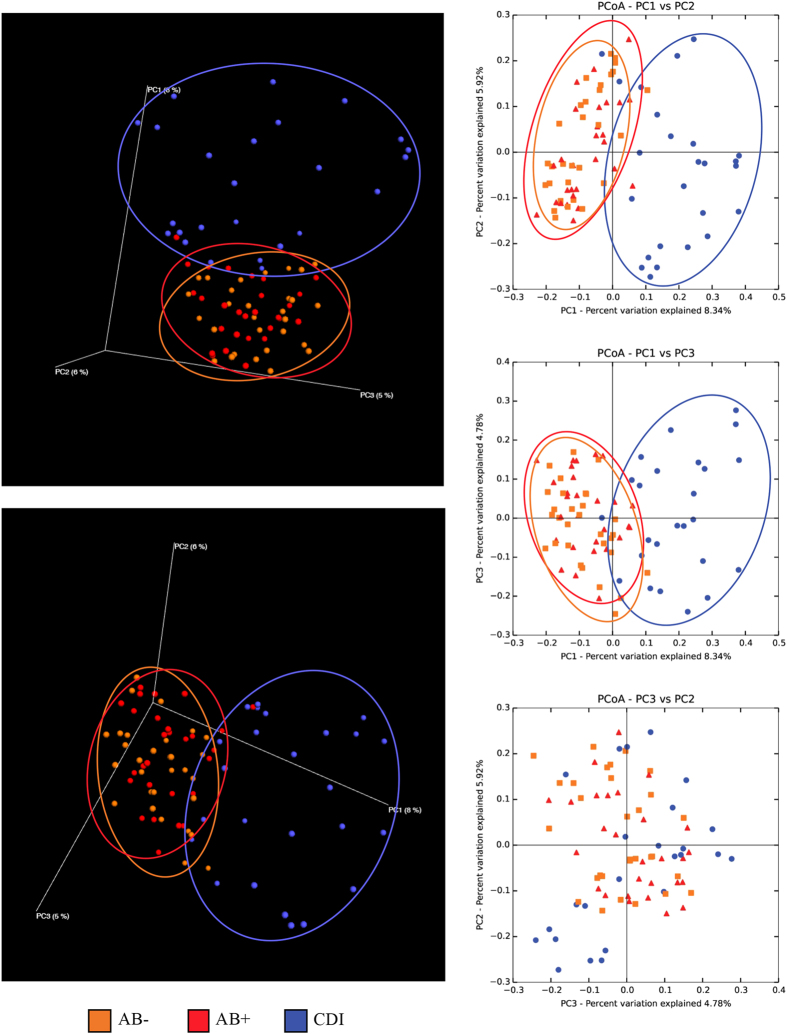
Evaluation of beta-diversity in AB−, AB+ and CDI samples. The predicted PCoA encompassing all 84 AB−, AB+ and CDI datasets is reported through two three-dimensional images as well as two-dimensional sections. AB−, AB+ and CDI datasets and corresponding clusters are colored in orange, red and blue, respectively.

The Principal Coordinate Analysis (PCoA) built based on unweighted UniFrac matrix and the beta diversity among the samples allowed a detailed analysis of the similarities between the gut microbiota composition of the different samples (Fig. 2). Despite high inter-sample variability across groups, AB− and AB+ appeared to cluster together, while CDI samples clustered separately (Fig. 2). Thus, the microbiota of CDI-affected individuals is characterized by remarkably higher differences in the overall composition respect to the microbiota of AB+ when both are compared to AB− subjects. This observation was also confirmed by p-value of PERMANOVA statistical analysis <0.001 obtained by comparison of CDI versus AB+ and AB− samples. Notably, the higher spread of CDI samples in PCoA representation indicated higher CDI group inter-sample variability compared to AB− and AB+ (Fig. 2).

### Comparison of the gut microbiota composition across different groups

Inspection of predicted taxonomic profiles at genus level for all samples, presented in Fig. S1, allowed the identification of 136 taxa with abundance >0.1% in at least one sample. Among them, 99, 99 and 84 were identified in AB−, AB+ and CDI groups, respectively (Fig. S1), confirming the reduced biodiversity in CDI samples observed through the alpha diversity analysis (Fig. 1).

**Figure 3:**
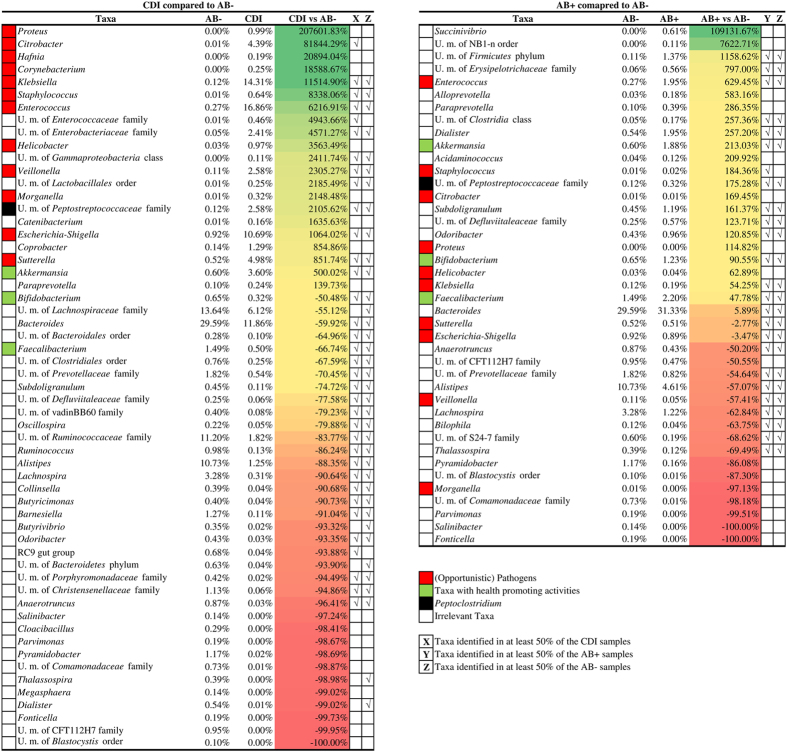
Impact of antibiotic treatments and CDI on the gut microbiota. Panel **a** displays a heat map illustrating bacterial taxa with mean relative abundance >0.1% and showing increase >100% or decrease <−50% in CDI compared to AB− datasets. Panel **b** shows a heat map illustrating bacterial taxa with mean relative abundance >0.1% and exhibiting an increase >100% or decrease <50% in datasets obtained from AB+ samples as compared to those from AB− samples. Taxa falling outside the cut-offs but being of particular relevance are also reported in the heat maps. Taxa detected in at least 50% of the sample constituting AB−, AB+ and CDI groups are marked.

In order to better identify differences in microbiota composition between AB−, AB+ and CDI groups, we focused on the 77 genera with an average relative abundance of >0.1% in at least one group (Table S2). Comparing AB− versus CDI, we found an overrepresentation greater than 100% for 17 taxa in the CDI group. Conversely, we found an underrepresentation (lower than 50%) for 41 taxa in the same group (Table S2 and Fig. 3). These underrepresented taxa included bacteria with purported health-promoting activities^38^,^39^,^40^, such as *Faecalibacterium, Bifidobacterium* and *Akkermansia* as well as other gut commensals typically abundant in a healthy microbiota such as *Bacteroides*, unclassified members of *Lachnospiraceae* family, unclassified members of *Ruminococcaceae* family and *Alistipes* (Fig. 3).

Moreover, in addition to an increase (21-fold) in unclassified members of Peptostreptococcaceae, encompassing *C. difficile* (Fig. 3), a plethora of pathogens or opportunistic pathogens were present at markedly higher mean relative abundance in CDI compared to AB− samples. Such genera included *Klebsiella, Escherichia/Shigella, Sutterella, Enterococcus, Citrobacter, Veillonella, Proteus, Helicobacter, Morganella, Hafnia, Corynebacterium* and *Staphylococcus.* All these taxa showed substantial overrepresentation compared to AB− subjects, ranging from 8- to 200-fold (Fig. 3).

At an age- and sex-adjusted statistical model, we identified 14 taxa, i.e., *Anaerotruncus, Barnesiella, Butyricimonas, Collinsella, Lachnospira, Alistipes, Oscillospira, Subdoligranulum, Bacteroides* and unclassified members of *Christensenellaceae* family, *Ruminococcaceae* family, *Clostridiales* order, *Bacteroidales* order and *Lachnospiraceae* family, significantly depleted (p < 0.05) in CDI compared to AB− group (Tables 2 and S2). In a statistical model accounting also for clinical variables that were significantly different between the two groups, i.e. number of medications and duration of hospital stay (Table 2), only five taxa were confirmed to be significantly depleted. These included *Butyricimonas* (p = 0.03), *Alistipes* (p = 0.03), *Oscillospira* (p = 0.008) as well as unclassified members of *Ruminococcaceae* (p = 0.003) and *Clostridiales* (p = 0.02). Conversely, a significant over-representation of *Klebsiella* (p = 0.003) as well as unclassified members of *Enterobacteriaceae* (p = 0.006) and *Gammaproteobacteria* (p = 0.03) was identified in CDI samples (Table 2).

On the other side, 17 taxa were underrepresented (<50%) in AB+ compared to AB− samples, including *Alistipes, Bilophila, Salinibacter* and *Fonticella*. Conversely, 22 taxa showed overrepresentation (>100%), including *Staphylococcus, Akkermansia, Enterococcus* and *Succinivibrio*.

In the statistical model adjusted for age, sex, number of medications and duration of hospital stay (Table 2), AB+ groups showed a significant underrepresentation of *Alistipes* (p = 0.03) and *Bilophila* (p = 0.04) compared to AB−. On the other side, no bacterial taxa showed a statistically significant overrepresentation in AB+ vs AB− group.

In order to identify key microbial biomarkers of high risk of CDI development (CDImb), we focused on the 12 bacterial taxa exhibiting average reduction lower than 50% and the 11 taxa showing average increase higher than 100% in both AB+ and CDI groups compared to AB− subjects (Table S2, taxa underlined). Among these, *Alistipes* mean relative abundance ranged from 10.73% in AB− to 1.25% in CDI subjects, while *Enterococcus* ranged from 0.27% in AB− to 16.86% in CDI datasets, thus representing the gut microbiota members showing the highest mean relative abundance variation in altered microbiota conditions linked to CDI (Table S2).

**Figure 4:**
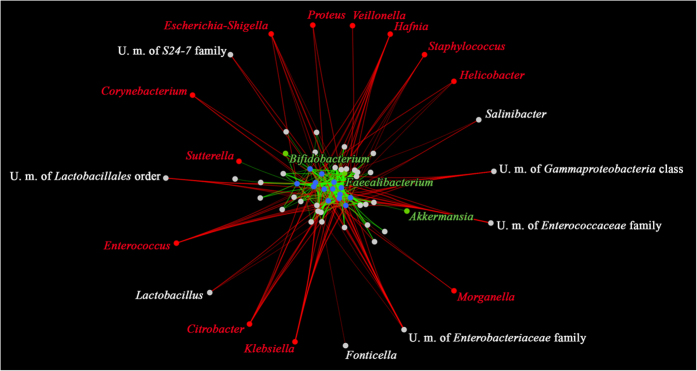
Role of CDImb in protection from gut microbiota alterations and opportunistic pathogens. The network representation shows the impact of CDImb (blue dots) on the whole microbiome. Links between the dots represent positive (in green) or negative (in red) correlations based on Kendall tau-rank co-variance analysis. Each link acts as a spring whose attracting or repulsing force as well as line width is proportional to the positive or negative co-variance values, respectively. Taxa with purported health-promoting activities are colored in green while opportunistic pathogens are colored in red.

All the 70 taxa with average relative abundance >0.1% in the AB− or CDI datasets were employed in co-variance analysis based on Kendall tau-rank. Co-variance datasets including CDImb were used to construct a force-driven network map (Table S2) (Fig. 4). This network displayed the extensive impact of the decrease of CDImb on the whole microbiota. Interestingly, opportunistic pathogens identified in CDI patients (Fig. 3) correlated negatively with the presence of CDImb (Fig. 4). In contrast, taxa known to exert beneficial effects on the host such as *Bifidobacterium, Faecalibacterium* and *Akkermansia* were positively correlated with the CDImb (Fig. 4).

### Microbiome functionality assessed by shotgun metagenomics

In order to further explore the impact of antibiotic exposure and *C. difficile* infection on microbiome functionality, we performed shotgun metagenomics of the microbial DNA extracted from fecal samples of 15 randomly selected samples (5 AB−, 5 AB+ and 5 CDI) (Table S3). NGS sequencing of these 15 samples produced a total of 7,907,174 raw reads that were filtered for human DNA and further cleaned up by quality control, resulting in 5,038,536 filtered reads that were used for the following analyses.

Reconstruction of the microbiome allowed functional classification of coding reads by means of the EggNog database^41^. This analysis showed a 21.7% overrepresentation in carbohydrate transport and metabolism in AB+ as compared to AB− datasets, and a 23.9% overrepresentation in CDI compared to AB− datasets (Fig. S2). In addition, compared to AB− subjects, the CDI data sets were characterized by a 14.8% expansion of the Cluster of Orthologous Genes (COG) family encompassing the amino acids transport and metabolism as well as a 12.2% expansion of the COG family corresponding to energy production and conversion (Fig. S2). Comparison of the detected glycosyl hydrolase (GH) profiles showed that CDI microbiomes had a wider arsenal of GHs of the GH68, GH70, GH4, GH1, GH63, GH32 and GH13 families compared to AB− subjects, with an overrepresentation ranging from 20% to 2345% (Fig. S2).

Taxonomic classification of reads corresponding to coding regions confirmed higher abundance of opportunistic pathogenic genera and lower abundance of microbial taxa known to be linked to a healthy microbiota in CDI samples, such as *Bifidobacterium, Akkermansia* and *Faecalibacterium*^38^,^39^,^40^ (Fig. S2). An assessment of bacterial antibiotic resistance genes, encompassing both enzymes and transporter systems showed an overall overrepresentation of antibiotic resistance genes counts in CDI (32.16%) and AB+ (0.95%) compared to AB− samples (Fig. S2).

Moreover, analysis of predicted bacterial metabolic pathways showed a 53.1% enhancement in genes involved in succinate production in CDI compared to AB− microbiomes (Fig. S2). Since *C. difficile* metabolism of succinate lead to production of butyrate, we also evaluated the modulation of pathways involved in production of this Short Chain Fatty Acid (SCFA). Notably, *in silico* analyses of the CDI and AB− microbiomes revealed a 449.7% overrepresentation of genes encompassing the butyrate biosynthesis pathway in CDI compared to AB− datasets (Fig. S2).

Furthermore, we evaluated the presence of pathways involved in bile salt de-conjugation and primary bile acid conversion to secondary bile acids in the CDI and AB− microbiomes. Notably, pathway abundance data showed a 17.9% reduction of the glycocholate metabolism and a 57.3% reduction of the cholate degradation (Fig. S2). Furthermore, underrepresentation of the glycocholate metabolism pathway by 48.4% has also been observed comparing AB+ and AB− metagenomics datasets.

### Detailed characterization of the Peptostreptococcaceae community

Mapping of the shotgun metagenomics reads against available genomes from members of the Peptostreptococcaeae family allowed a detailed profiling of the Peptostreptococcaceae population in AB−, AB+ and CDI samples (Fig. S3). The mean relative abundance of the Peptostreptococcaceae community differed in AB− and CDI subjects, being 0.18% and 0.41% of the whole microbiota composition, respectively (Fig. S3). Notably, the mean relative abundance of pathogenic clostridia, as compared to the overall Peptostreptococcaceae population, ranged from 37% in AB− to 79% in CDI subjects, with simultaneous reduction of non-pathogenic commensals *Romboutsia* sp. and *Terrisporobacter glycolicus*^42^. The commensal/opportunistic pathogen ratio was also decreased (Fig. S3).

## Discussion

In a group of multimorbid elderly patients following a standard diet, hospitalized for extra-intestinal diseases, *Clostridium difficile* infection was associated with peculiar features of fecal microbiota compared to *C. difficile* negative patients. These features included reduced microbial population complexity, significantly different taxonomic profiles and high inter-individual variability. At genus level, some putative protective components of microbiota, like *Alistipes, Butyricimonas* and *Oscillospira*, were significantly underrepresented in CDI samples. These taxa can be of high relevance for the definition of putative microbial biomarkers of CDI (CDImb) through comparison and integration with similar studies involving various human populations as well as standardized mouse models. On the other side, a large number of opportunistic pathogens were overrepresented in CDI samples.

*Clostridium difficile*-negative patients exposed to antibiotic treatment instead did not display a significantly different taxonomic profile of the overall fecal microbial population compared to subjects unexposed to antibiotics. However, AB+ showed significant underrepresentation of some key taxa, like *Alistipes*.

Successful gut colonization by *C. difficile* has been associated with antibiotic treatment and alterations of the gut microbiota in both humans and mouse models. These alterations may be affected by the type and dose of antibiotic treatment^6^,^11^,^12^. Nevertheless, key microbial taxa that might be critical for protection against CDI have not yet been fully identified, particularly in a high-risk elderly population. To date, only one metagenomic study has shown the fecal microbiota composition in this setting^43^. However, a very high inter-individual variability, possibly due to different setting of living of the enrolled patients (e.g., community, rehabilitation facilities, nursing homes, hospital), prevented solid conclusions to be drawn from that particular study.

In the here presented hospital-based study, all enrolled patients were similar in terms of age, weight, multimorbidity burden as well as functional status and they followed the same hospital-based oral diet. They differed only for CDI, number of administered drugs, duration of hospital stay and exposure to antibiotics. As such, since prolonged hospital stay and antibiotic exposure are the main risk factors for nosocomial CDI onset, the three groups of our study may represent three different stages of the same physio-pathological continuum. The AB− group encompassed acutely-ill elderly with a low risk of CDI and thus at the beginning of the physio-pathological cascade. AB+ patients instead had a high risk of contracting CDI, as they were exposed to antibiotics. The differences in the composition of their fecal microbiota may help us to understand how these drugs impact on gut homeostasis, predisposing to a possible ensuing onset of CDI. Finally, CDI patients were at the end of the physio-pathological cascade, as they already suffered from disease. Notably, the comparison of CDI vs AB+ gut microbiomes can help to understand the mechanisms that lead to loss of colonization resistance to *C. difficile*.

As such, the antibiotic treatment may have possible long-term negative effects in terms of stability of the gut microbiota. Our analyses highlighted the existence of 14 bacterial taxa, whose reduction in mean relative abundance was associated with the presence of CDI. We may speculate that such taxa (CDImb) represent early markers of the development of CDI. This is in line with the ecological theory of tipping elements that indicate the change to alternative stable states of the gut microbiota, for which experimental evidences are growing^44^. Notably, statistical analysis of CDI subjects compared to both AB+ and AB− showed the possible role in the development of microbiota deviations exerted by two potential CDImb, *Alistipes* and *Enterococcus*, which were under- and over-represented, respectively. Thus, we can hypothesize that changes in the mean relative abundance of these taxa may represent an early event in the physio-pathological cascade leading to loss of *C. difficile* colonization resistance. According to this theory, *Alistipes* could represent a possible future candidate for preventive or curative next-generation bacterial therapy, by antagonizing the deleterious effects of prolonged antibiotic treatment. Interestingly, *Alistipes* under-representation in gut microbiota has recently been associated with antibiotic treatment and increased susceptibility of CDI in a mouse model, supporting its role as a biomarker of CDI (CDImb)^45^.

As hypothesized by co-occurrence analysis, reduction of CDImb and associated gut microbiota alterations may allow colonization by a wide array of opportunistic pathogens, including *C. difficile*. The colonization of these opportunistic pathogens may be a consequence of the simplification of the gut microbiota composition and the accessibility of ecological niches that under normal conditions are occupied by depleted CDImb. In CDI samples, the overrepresentation of opportunistic pathogens, such as *Klebsiella* and members of *Enterobacteriaceae,* may simply reflect a “blooming” phenomenon, due to a reduced ecological niche competition^46^. Even the relative overrepresentation of some taxa that are associated with health and improving barrier function, like *Bifidobacterium* and *Akkermansia* spp^47^,^48^,^49^,^50^, seen within some of the CDI samples, could reflect an epi-phenomenon due to modifications in the gut micro-environment. For example, *Akkermansia* over-representation may reflect enteric mucosa inflammation with increased mucus production^51^.

Microbiome resilience to a perturbative event, such as antibiotic administration, may thus be crucial for homeostasis maintenance and protection against CDI^52^. Prolonged antibiotic exposure, similar to that seen in our CDI group, may overwhelm such microbiome resilience and result in more evident aberrations. Unfortunately, a reduced microbiome resilience is also associated with the ageing process per se, and this may explain why the elderly have a particularly high risk of developing CDI^19^. Notably, the presence of frailty, multimorbidity and institutionalization is associated with reduced microbial diversity resulting in an increased susceptibility to opportunistic infections^20^,^21^,^22^.

Furthermore, functional characterization of the microbiome of CDI subjects allowed the identification of changes in the microbiome, such as increase of GHs and other genes constituting pathways involved in glucose, fructose, succinate and butyrate production as well as reduction of genes responsible for primary bile acid degradation. These observations are consistent with previously published *in vitro* and metabolomic data regarding environmental factors that favor CDI onset^53^,^54^. Such factors include a reduction of secondary bile acids as well as an increase of primary bile acids, succinate and sugar alcohols^53^,^54^. Moreover, these studies reported the ability of *C. difficile* to exploit primary bile acids for germination and carbon sources such as succinate or simple sugars like fructose for optimal growth in the gastrointestinal-tract^53^,^54^. Notably, a shift in the occurrence of certain members of the Peptostreptococcaceae family toward an enrichment of pathogenic clostridia and a decrease of non-pathogenic commensals, including *Romboutsia* sp. and *Terrisporobacter glycolicus*^42^, was observed in CDI patients. The reduction of these non-pathogenic Peptostreptococcaceae species might thus represent an event that triggers the blooming of *C. difficile* at the very early stages of infection.

However, some limitations should be considered when interpreting our results. In fact, the cross-sectional study design did not allow to establish whether each of the detected alterations in the gut microbiota of CDI patients was a cause or a consequence of *C. difficile* infection. Furthermore, hospital length of stay and drug treatment were not comparable among the three groups. However, the microbiomes of AB+ patients clustered together irrespective of duration of antibiotic exposure, thus suggesting that this element had minimal influence on overall gut microbial ecology. In addition, the clinical complexity of patients and the need of polypharmacy did not allow us to standardize antibiotic treatment and to identify a sufficient number of samples for each type of drug.

However, these limitations are offset by considerable strenghts. To our knowledge, this is the first study exploring the composition of fecal microbiota using a metagenomic approach in a hospitalized multimorbid elderly population with CDI or different degrees of CDI risk under standard dietary regimen. The high sequencing depth and accuracy of metagenomic techniques applied in this study allowed us to obtain reliable data on complex phenomena such as differences in microbial ecology. Finally, the presence of a large set of clinical data allowed us to perform statistical adjustment of results for covariates.

## Conclusions

In a cohort of hospitalized multimorbid elderly patients under standard oral diet, *Clostridium difficile* infection was associated with gut microbiota alterations, including significant depletion of some putative protective taxa and overrepresentation of opportunistic pathogens. Antibiotic exposure in *Clostridium difficile*-negative patients was instead associated with subtler alterations. The CDI microbial biomarkers identified in our study group, like *Alistipes*, might allow in pivotal future studies a better understanding of the physiopathology of CDI and, possibly, the development of novel bacterial therapies antagonizing gut microbiota alterations.

## Additional Information

**How to cite this article**: Milani, C. *et al*. Gut microbiota composition and *Clostridium difficile* infection in hospitalized elderly individuals: a metagenomic study. *Sci. Rep.* **6**, 25945; doi: 10.1038/srep25945 (2016).

## Supplementary Material

Supplementary Information

## Figures and Tables

**Table 1:** Clinical characteristics of the studied population (n = 84).

	CDI	AB+	AB−	p*
	(n = 25)	(n = 29)	(n = 30)	
Age (years)	82.9 ± 8.5	84.2 ± 8.1	82.3 ± 6.8	0.56
Weight	63.9 ± 15.9	68.4 ± 20.9	72.0 ± 15.1	0.29
CIRS Severity Index	2.5 ± 1.3	2.0 ± 1.3	2.0 ± 1.2	0.11
Rockwood Frailty Index	5.6 ± 1.6	5.4 ± 1.9	5.2 ± 1.8	0.68
Number of Drugs	14.6 ± 5.3	11.7 ± 3.3	8.0 ± 2.8	<.0001
PPI exposure	64	82	65	0.73
Days of hospitalization	31 [18–53]	9 [5.0–24]	6 [4–14]	<.0001
Days of antibiotic exposure	8 [1–17]	3 [2–7.5]	–	0.007
Levofloxacin exposure	56	45	–	0.06
Ceftriaxone exposure	44	41	–	0.56
Azithromycin exposure	16	38	–	0.87
Piperacillin/tazobactam exposure	60	24	–	0.007
Ampicillin exposure	12	17	–	0.50

(CDI: *Clostridium difficile* infected samples, n = 25; AB+: *Clostridium difficile*-negative samples, patients exposed to antibiotic treatment, n = 29; AB−: *Clostridium difficile*-negative samples, patients not exposed to antibiotic treatment, n = 30). Data are presented as mean ± standard deviation, median [IQR] or percentage where appropriate.

^*^Age- and sex-adjusted (where appropriated), Kruskal-Wallis test or chi-square test.

**Table 2:** Comparison of the mean relative abundance in fecal microbiota of a selection of taxa among the three study groups (CDI, AB+ and AB−).

Taxa#,♦	AB−	AB+	CDI	AB+ vs AB−	p-value*	p-value°	CDI vs AB+	p-value*	p-value°	CDI vs AB−	p-value*	p-value°
*Salinibacter*	0.14%	0.00%	0.00%	−100.00%	0.75		–	0.01	0.05	−97.24%	0.47	
*Anaerotruncus*	0.87%	0.43%	0.03%	−50.20%	0.07		−92.79%	0.06		−96.41%	0.03	0.16
U. m. of *Christensenellaceae* family	1.13%	2.08%	0.06%	83.59%	0.48		−97.20%	0.06		−94.86%	0.006	0.11
U. m. of *Porphyromonadaceae* family	0.42%	0.27%	0.02%	−36.72%	0.76		−91.29%	0.03	0.08	−94.49%	0.09	
*Barnesiella*	1.27%	1.03%	0.11%	−18.73%	0.68		−88.97%	0.02	0.08	−91.04%	0.01	0.10
*Butyricimonas*	0.40%	0.48%	0.04%	21.01%	0.95		−92.34%	0.02	0.009	−90.73%	0.001	0.03
*Collinsella*	0.39%	0.26%	0.04%	−33.80%	0.4		−85.93%	0.006	0.02	−90.68%	0.03	0.19
*Lachnospira*	3.28%	1.22%	0.31%	−62.84%	0.23		−74.81%	0.19		−90.64%	0.005	0.06
*Alistipes*	10.73%	4.61%	1.25%	−57.07%	0.001	0.03	−72.87%	0.06		−88.35%	0.0002	0.03
U. m. of *Ruminococcaceae* family	11.20%	9.76%	1.82%	−12.81%	0.29		−81.39%	0.002	0.01	−83.77%	<0.001	0.003
*Oscillospira*	0.22%	0.38%	0.05%	67.97%	0.1		−88.02%	0.0004	0.0009	−79.88%	0.0002	0.008
*Subdoligranulum*	0.45%	1.19%	0.11%	161.37%	0.21		−90.33%	0.04	0.09	−74.72%	0.03	0.13
U. m. of *Clostridiales* order	0.76%	1.32%	0.25%	74.93%	0.13		−81.47%	0.008	0.004	−67.59%	0.001	0.02
U. m. of *Bacteroidales* order	0.28%	0.24%	0.10%	−12.77%	0.3		−59.83%	0.07		−64.96%	0.02	0.39
*Bacteroides*	29.59%	31.33%	11.86%	5.89%	0.61		−62.15%	0.002	0.01	−59.92%	0.004	0.07
U. m. of *Lachnospiraceae* family	13.64%	11.81%	6.12%	−13.37%	0.74		−48.20%	0.09		−55.12%	0.04	0.29
*Bilophila*	0.12%	0.04%	0.09%	−63.75%	0.02	0.04	105.74%	0.56		−25.42%	0.67	
*Escherichia-Shigella*	0.92%	0.89%	10.69%	−3.47%	0.6		1105.91%	0.55		1064.02%	0.006	0.45
U. m. of *Lactobacillales* order	0.01%	0.05%	0.25%	394.86%	0.07		361.84%	0.07		2185.49%	0.005	0.21
U. m. of *Gammaproteobacteria* class	0.00%	0.01%	0.11%	144.16%	0.38		928.75%	0.02	0.04	2411.74%	0.002	0.03
*Helicobacter*	0.03%	0.04%	0.97%	62.89%	0.9		2149.13%	0.02	0.04	3563.49%	0.03	0.17
U. m. of *Enterobacteriaceae* family	0.05%	0.06%	2.41%	20.68%	0.84		3770.70%	0.02	0.006	4571.27%	0.009	0.006
U. m. of *Enterococcaceae* family	0.01%	0.03%	0.46%	192.58%	0.11		1623.84%	0.008	0.09	4943.66%	0.02	0.38
*Enterococcus*	0.27%	1.95%	16.86%	629.45%	0.03	0.07	765.98%	0.0008	0.03	6216.91%	0.0005	0.13
*Klebsiella*	0.12%	0.19%	14.31%	54.25%	0.48		7429.84%	0.008	0.004	11514.90%	0.002	0.003

Only taxa with a significant age- and sex-adjusted difference in at least one of the three comparisons (AB+ vs AB−, CDI vs AB+, CDI vs AB−) are reported in this Table. The full Table, including all taxa with an average relative abundance >0.1% in at least one group (77 taxa), is available in Supplemental Material. (CDI: *Clostridium difficile* infected samples, n = 25; AB+: *Clostridium difficile*-negative samples, patients exposed to antibiotic treatment, n = 29; AB−: *Clostridium difficile*-negative samples, patients not exposed to antibiotic treatment, n = 30).

U.m. = Unclassified members.

^*^Age- and sex-adjusted.

^°^Fully-adjusted (age, sex, length of hospital stay, number of drugs).

^#^Taxa in bold show decrease lower than 50% in CDI vs AB− subjects with p-value < 0.005.

^♦^Taxa underlined show decrease lower than 50% or increase higher than 100% in AB and CDI vs AB− subjects.
