# Supplementary material for: Gut microbiota composition and Clostridium difficile infection in hospitalized elderly individuals: a metagenomic study
Source: Sci Rep. 2016 May 11;6:25945. doi: 10.1038/srep25945 (PMC4863157; doi:10.1038/srep25945)
Supplement: Supplementary Information [file srep25945-s1.pdf]

Gut microbiota composition and *Clostridium difficile* infection in hospitalized elderly individuals: a metagenomic study

Christian Milani[1#], Andrea Ticinesi[2#], Jacoline Gerritsen[3], Antonio Nouvenne[2], Gabriele Andrea Lugli[1], Leonardo Mancabelli[1], Francesca Turroni[1], Sabrina Duranti[1], Marta Mangifesta[1], Alice Viappiani[4], Chiara Ferrario[1], Marcello Maggio[2], Fulvio Lauretani[2], Willem De Vos[3], Douwe van Sinderen[5], Tiziana Meschi[2]*, Marco Ventura[1]*

Supplementary information

Supplementary figures legends

Figure S1: Taxonomic profiles of the 84 analyzed 16S rRNA profiling datasets. The bar plot shows taxonomy profiles at genera level of taxa with relative abundance >1 %. AB-, AB+ and CDI datasets are grouped separately. (CDI: *Clostridium difficile* infected samples; AB+: *Clostridium difficile*-negative samples, patients exposed to antibiotic treatment; AB-: *Clostridium difficile*-negative samples, patients not exposed to antibiotic treatment).

Figure S2. Functional changes in the gut microbiome of AB+ and CDI compared to AB- subjects, part 1. Panel a shows relative abundance of *Clostridium difficile* microbial biomarkers (CDImb) in AB-, AB+ and CDI shotgun metagenomic datasets. Panel b reports the relative abundance of taxa with supposed health-promoting activities and opportunistic pathogens in AB-, AB+ and CDI samples. Panel c illustrates percentage variation in abundance of enzymes and transporters involved in antibiotic resistance in AB+ and CDI compared to AB- subjects as well as CDI compared to AB+ subjects. Panel d depicts variations of abundance of Cluster of Orthologous Genes (COG) functional families involved in energy production in AB+ and CDI compared to AB- samples. COG functional families are abbreviated as follows: [C] Energy production and conversion, [G] Carbohydrate transport and metabolism, [E] Amino acid transport and metabolism. Panel e shows changes in Glycosyl Hydrolase (GH) families involved in glucose (circled in green) and fructose (circled in purple) production in AB+ and CDI compared to AB- datasets. Panel f exhibits variation in bile salts and bile acid degradation pathways in AB+ and CDI compared to AB- samples as well as CDI compared to AB+ subjects. Panel g displays variation of pathways involved in butyrate production in AB+ and CDI compared to AB- samples as well as CDI compared to AB+ subjects. Panel h shows variation of pathways involved in succinate production in AB+ and CDI compared to AB- samples as well as CDI compared to AB+

subjects. (CDI: *Clostridium difficile* infected samples; AB+: *Clostridium difficile*-negative samples, patients exposed to antibiotic treatment; AB-: *Clostridium difficile*-negative samples, patients not exposed to antibiotic treatment).

Figure S3. Profiling of *Peptostreptococcaceae* population based on shotgun metagenomics data in AB-, AB+ and CDI samples. Panel a shows cake diagrams depicting the taxonomic composition at species level of the AB-, AB+ and CDI datasets. Panel b displays bar plots presenting the variation in relative abundance of the mapped *Peptostreptococcaceae* species in the AB-, AB+ and CDI subjects. Panel c illustrates, by means of bar plots, the variation in the commensals/(opportunistic) pathogens ratio observed for the *Peptostreptococcaceae* family population in AB-, AB+ and CDI datasets. (CDI: *Clostridium difficile* infected samples; AB+: *Clostridium difficile*-negative samples, patients exposed to antibiotic treatment; AB-: *Clostridium difficile*-negative samples, patients not exposed to antibiotic treatment)

Table S1. Filtering report of 16S rRNA profiling datasets.

Sample	Number of reads	Number of reads removed because of:					Reverse primers not found	Final reads number	average reads length
		Outside bounds (100-400)	Ambiguous bases	Mean quality <25	Homopolymer runs >7bp	Primer mismatch >1			
1AB-	120015	25611	0	15844	296	23462	13018	41784	187.4
2AB-	150273	34468	0	15725	648	14499	28779	56153	182.7
3AB-	90574	15984	0	7645	267	11012	9389	46275	188.9
4AB-	91739	22463	0	10853	787	6346	17404	33882	181.4
5AB-	160563	35110	0	16634	36	15115	17926	75742	188.3
6AB-	77214	10825	0	8957	131	9232	10356	37713	192.1
7AB-	86507	10776	0	11486	173	16364	16829	30878	188.8
8AB-	97352	28937	0	9087	160	6649	14244	38273	182.5
9AB-	162000	45484	0	18677	639	13246	23667	60287	181.7
10AB-	123875	25812	0	11418	893	10878	15651	59223	183.1
11AB-	114148	28437	0	16763	131	7335	18555	42927	175.6
12AB-	147650	60398	0	9197	1490	10233	18371	47961	169.2
13AB-	194079	64295	0	58643	87	7484	13898	49672	170.2
14AB-	178628	65987	0	21501	83	16174	20809	54074	157.8
15AB-	122748	38119	0	17612	272	7774	15756	43215	176.3
16AB-	89617	21741	0	9565	158	9297	8347	40509	189.8
17AB-	124480	35139	0	10292	65	8725	24926	45333	170.2
18AB-	129985	36348	0	8056	43	9998	27675	47865	168.4
19AB-	212917	61536	0	18144	670	10070	27260	95237	170.6
20AB-	90294	30311	0	7886	310	4862	14675	32250	167.9
21AB-	147459	49699	0	9530	111	13506	20887	53726	171
22AB-	112246	42991	0	10420	819	6148	17191	34677	167
23AB-	87547	18622	0	4511	250	5299	7610	51255	173
24AB-	132896	46046	0	9815	484	11019	16466	49066	172.5
25AB-	167416	48641	0	15847	298	15386	26977	60267	168.5
26AB-	145410	34350	0	17439	136	11172	32881	49423	181.9
27AB-	173005	48307	0	11260	749	9762	17432	85495	186.7
28AB-	77540	11782	0	7957	189	4520	11743	41349	190.3
29AB-	118190	24809	0	11886	157	9233	14895	57210	191.4
30AB-	92259	20246	0	8127	150	8940	14568	40228	190
1AB+	124659	23180	0	16251	176	10894	20302	53856	174.6
2AB+	82771	19963	0	13018	119	6166	24534	18970	174.2
3AB+	216471	83802	0	13894	382	21343	33787	63263	170.9
4AB+	126768	47446	0	22817	179	7681	10611	38034	167.9
5AB+	138992	39084	0	21111	95	12274	22720	43708	172.3
6AB+	119878	19465	0	13670	194	11898	39634	35017	186
7AB+	184040	55783	0	48059	24	10458	24976	44740	169.4
8AB+	73703	19219	0	4568	437	4642	13249	31588	168.4
9AB+	105604	26873	0	6429	191	6331	20966	44813	168.2
10AB+	99644	19952	0	10856	131	8722	6668	53315	177.1
11AB+	114953	30041	0	11576	60	7386	12726	53156	178.8
12AB+	95181	20791	0	8336	121	7714	6249	51970	180.3
13AB+	58793	25459	0	3728	77	3001	5254	21274	170.8

14AB+	192800	68806	0	12214	1236	11039	26415	73090	167.6
15AB+	155536	45816	0	40475	141	7015	32043	30046	163.5
16AB+	121227	52190	0	7261	1190	3747	14700	42139	165.3
17AB+	162464	65499	0	11420	337	11104	19654	54450	169.7
18AB+	148437	46913	0	8493	405	10080	22871	59675	169.7
19AB+	96451	41524	0	4608	237	5422	11571	33089	168.9
20AB+	193373	42968	0	10981	275	15926	18517	104706	179.4
21AB+	125361	28892	0	6566	287	9754	14351	65511	178.4
22AB+	129020	38532	0	8335	262	9598	20093	52200	172.8
23AB+	116035	39367	0	12692	238	6669	22268	34801	169.7
24AB+	170079	59554	0	15168	1210	11587	23097	59463	167.8
25AB+	139482	54150	0	10116	183	11330	23721	39982	167.5
26AB+	152175	54927	0	16274	194	5879	27817	47084	165.3
27AB+	56425	8284	0	5769	39	6446	13751	22136	188.8
28AB+	146431	49403	0	11290	243	10922	30965	43608	170.4
29AB+	87228	17121	0	8685	163	8697	11812	40750	187.5
1CDI	146627	27395	0	17164	41	21820	28161	52046	182.6
2CDI	158053	32477	0	12869	36	15843	11433	85395	180
3CDI	108358	22376	0	13727	75	9639	30481	32060	167
4CDI	91899	28451	0	11783	49	9095	15969	26552	175.4
5CDI	152213	22158	0	17148	107	20023	53099	39678	165.7
6CDI	115112	19433	0	34021	44	9265	35326	17023	157.1
7CDI	182926	36499	0	25893	60	14135	47332	59007	174.8
8CDI	145673	28459	0	11771	135	15740	38029	51539	174.3
9CDI	101190	25146	0	8822	167	5687	17050	44318	173.2
10CDI	171412	24157	0	11967	131	24002	25385	85770	174.3
11CDI	128997	38193	0	8355	191	13205	18988	50065	171.5
12CDI	84573	23951	0	15418	205	3300	12209	29490	173.5
13CDI	132382	38732	0	13046	256	9706	34317	36325	172.6
14CDI	123579	29681	0	14228	111	10285	41162	28112	169
15CDI	184050	58818	0	10763	493	15846	24403	73727	177.1
16CDI	146186	16720	0	17018	127	21730	36819	53772	186.9
17CDI	112366	34677	0	27145	38	7643	25841	17022	167
18CDI	138705	21590	0	15177	138	17018	23796	60980	187.8
19CDI	111768	29306	0	10762	87	10982	23976	36655	185.9
20CDI	11960	0	0	0	1	236	6	11717	191.6
21CDI	31429	0	0	1	1	843	8	30576	193.2
22CDI	34375	0	0	0	4	1045	9	33317	190.8
23CDI	33355	0	0	1	2	837	9	32506	190.5
24CDI	19737	0	0	0	4	348	9	19376	188.6
25CDI	39032	0	0	0	1	727	14	38290	194.5

Table S2. Variations of taxa (> 0.1 %) between AB-, AB+ and CDI averages.

Taxa°✦	AB-	AB+	CDI	AB+ vs AB-	P-value*	CDI vs AB+	P-value*	CDI vs AB-	P-value*
U. m. of *Blastocystis* order	0.10%	0.01%	0.00%	-87.30%	0.21	-100.00%	0.18	-100.00%	0.23
U. m. of CFT112H7 family	0.95%	0.47%	0.00%	-50.55%	0.39	-99.90%	0.41	-99.95%	0.13
Fonticella	0.19%	0.00%	0.00%	-100.00%	0.55	-	0.13	-99.73%	0.3
Dialister	0.54%	1.95%	0.01%	257.20%	0.76	-99.73%	0.47	-99.02%	0.11
Megasphaera	0.14%	0.22%	0.00%	55.35%	0.61	-99.37%	0.28	-99.02%	0.19
Thalassospira	0.39%	0.12%	0.00%	-69.49%	0.23	-96.66%	0.7	-98.98%	0.17
U. m. of *Comamonadaceae* family	0.73%	0.01%	0.01%	-98.18%	0.52	-37.66%	0.61	-98.87%	0.3
Pyramidobacter	1.17%	0.16%	0.02%	-86.08%	0.27	-90.58%	0.39	-98.69%	0.3
Parvimonas	0.19%	0.00%	0.00%	-99.51%	0.15	174.62%	0.22	-98.67%	0.59
Cloacibacillus	0.29%	0.34%	0.00%	17.19%	0.95	-98.64%	0.29	-98.41%	0.28
Salinibacter	0.14%	0.00%	0.00%	-100.00%	0.75	-	0.01	-97.24%	0.47
Anaerotruncus	0.87%	0.43%	0.03%	-50.20%	0.07	-92.79%	0.06	-96.41%	0.03
**U. m. of *Christensenellaceae* family**	1.13%	2.08%	0.06%	83.59%	0.48	-97.20%	0.06	-94.86%	0.006
U. m. of *Porphyromonadaceae* family	0.42%	0.27%	0.02%	-36.72%	0.76	-91.29%	0.03	-94.49%	0.09
U. m. of *Bacteroidetes* phylum	0.63%	0.62%	0.04%	-2.67%	0.92	-93.73%	0.19	-93.90%	0.27
RC9 gut group	0.68%	0.58%	0.04%	-14.64%	0.87	-92.83%	0.28	-93.88%	0.25
U. m. of *Bacteria* kingdom	1.02%	2.12%	0.06%	107.36%	0.4	-96.98%	0.2	-93.74%	0.11
Odoribacter	0.43%	0.96%	0.03%	120.85%	0.33	-96.99%	0.06	-93.35%	0.09
Butyrivibrio	0.35%	0.19%	0.02%	-46.55%	0.62	-87.51%	0.19	-93.32%	0.29
Barnesiella	1.27%	1.03%	0.11%	-18.73%	0.68	-88.97%	0.02	-91.04%	0.01
Butyricimonas	0.40%	0.48%	0.04%	21.01%	0.95	-92.34%	0.02	-90.73%	0.001
Collinsella	0.39%	0.26%	0.04%	-33.80%	0.4	-85.93%	0.006	-90.68%	0.03
Lachnospira	3.28%	1.22%	0.31%	-62.84%	0.23	-74.81%	0.19	-90.64%	0.005
Alistipes	10.73%	4.61%	1.25%	-57.07%	0.001	-72.87%	0.06	-88.35%	0.0002
Acidaminococcus	0.04%	0.12%	0.01%	209.92%	0.42	-95.69%	0.14	-86.64%	0.34
Ruminococcus	0.98%	1.51%	0.13%	54.71%	0.28	-91.10%	0.06	-86.24%	0.17
**U. m. of *Ruminococcaceae* family**	11.20%	9.76%	1.82%	-12.81%	0.29	-81.39%	0.002	-83.77%	<,0001
U. m. of Family XIII family	0.10%	0.16%	0.02%	66.28%	0.52	-89.70%	0.32	-82.87%	0.22
U. m. of *Clostridia* class	0.05%	0.17%	0.01%	257.36%	0.64	-94.63%	0.42	-80.82%	0.17
Oscillospira	0.22%	0.38%	0.05%	67.97%	0.1	-88.02%	0.0004	-79.88%	0.0002
U. m. of vadinBB60 family	0.40%	0.67%	0.08%	66.51%	0.78	-87.52%	0.31	-79.23%	0.18
U. m. of *Defluviitaleaceae* family	0.25%	0.57%	0.06%	123.71%	0.2	-89.98%	0.28	-77.58%	0.4
U. m. of NB1-n order	0.00%	0.11%	0.00%	7622.71%	0.12	-99.69%	0.14	-76.44%	0.07
Subdoligranulum	0.45%	1.19%	0.11%	161.37%	0.21	-90.33%	0.04	-74.72%	0.03
U. m. of *Prevotellaceae* family	1.82%	0.82%	0.54%	-54.64%	0.53	-34.85%	0.77	-70.45%	0.27
**U. m. of *Clostridiales* order**	0.76%	1.32%	0.25%	74.93%	0.13	-81.47%	0.008	-67.59%	0.001
Faecalibacterium	1.49%	2.20%	0.50%	47.78%	0.34	-77.49%	0.11	-66.74%	0.16
**U. m. of *Bacteroidales* order**	0.28%	0.24%	0.10%	-12.77%	0.3	-59.83%	0.07	-64.96%	0.02
Bacteroides	29.59%	31.33%	11.86%	5.89%	0.61	-62.15%	0.002	-59.92%	0.004
**U. m. of *Lachnospiraceae* family**	13.64%	11.81%	6.12%	-13.37%	0.74	-48.20%	0.09	-55.12%	0.04
Bifidobacterium	0.65%	1.23%	0.32%	90.55%	0.49	-74.01%	0.14	-50.48%	0.2

Alloprevotella	0.03%	0.18%	0.02%	583.16%	0.46	-90.95%	0.25	-38.19%	0.76
Desulfovibrio	0.16%	0.25%	0.11%	55.23%	0.45	-58.12%	0.34	-34.99%	0.63
Parabacteroides	1.87%	2.73%	1.24%	46.08%	0.25	-54.65%	0.31	-33.76%	0.34
Phascolarctobacterium	0.38%	0.67%	0.25%	77.57%	0.09	-62.11%	0.18	-32.71%	0.43
Streptococcus	0.30%	0.20%	0.20%	-31.67%	0.85	-0.74%	0.83	-32.18%	0.64
Bilophila	0.12%	0.04%	0.09%	-63.75%	0.02	105.74%	0.56	-25.42%	0.67
Intestinimonas	0.37%	0.60%	0.27%	65.24%	0.99	-54.62%	0.42	-25.01%	0.28
U. m. of S24-7 family	0.60%	0.19%	0.49%	-68.62%	0.08	162.98%	0.15	-17.48%	0.77
U. m. of *Firmicutes* phylum	0.11%	1.37%	0.09%	1158.62%	0.35	-93.32%	0.41	-15.86%	0.91
Lactobacillus	0.72%	0.47%	0.68%	-34.81%	0.88	44.51%	0.81	-5.80%	0.59
Blautia	0.60%	0.85%	0.58%	42.71%	0.15	-31.39%	0.49	-2.08%	0.69
U. m. of *Erysipelotrichaceae* family	0.06%	0.56%	0.07%	797.00%	0.16	-87.69%	0.28	10.40%	0.63
Prevotella	1.82%	1.68%	2.66%	-7.55%	0.78	58.27%	0.84	46.32%	0.51
U. m. of *Clostridiaceae* 1 family	0.11%	0.15%	0.20%	35.45%	0.93	35.25%	0.82	83.20%	0.48
Paraprevotella	0.10%	0.39%	0.24%	286.35%	0.25	-37.95%	0.76	139.73%	0.48
Succinivibrio	0.00%	0.61%	0.00%	109131.67%	0.8	-99.75%	0.21	169.83%	0.11
Akkermansia	0.60%	1.88%	3.60%	213.03%	0.08	91.68%	0.18	500.02%	0.16
Sutterella	0.52%	0.51%	4.98%	-2.77%	0.65	878.81%	0.19	851.74%	0.15
Coprobacter	0.14%	0.17%	1.29%	22.29%	0.35	680.83%	0.41	854.86%	0.38
Escherichia-Shigella	0.92%	0.89%	10.69%	-3.47%	0.6	1105.91%	0.55	1064.02%	0.006
Catenibacterium	0.01%	0.01%	0.16%	-33.58%	0.57	2513.07%	0.15	1635.63%	0.25
U. m. of *Peptostreptococcaceae* family	0.12%	0.32%	2.58%	175.28%	0.25	701.23%	0.08	2105.62%	0.13
Morganella	0.01%	0.00%	0.32%	-97.13%	0.19	78189.14%	0.2	2148.48%	0.24
U. m. of *Lactobacillales* order	0.01%	0.05%	0.25%	394.86%	0.07	361.84%	0.07	2185.49%	0.005
Veillonella	0.11%	0.05%	2.58%	-57.41%	0.69	5547.37%	0.39	2305.27%	0.49
U. m. of *Gammaproteobacteria* class	0.00%	0.01%	0.11%	144.16%	0.38	928.75%	0.02	2411.74%	0.002
Helicobacter	0.03%	0.04%	0.97%	62.89%	0.9	2149.13%	0.02	3563.49%	0.03
U. m. of *Enterobacteriaceae* family	0.05%	0.06%	2.41%	20.68%	0.84	3770.70%	0.02	4571.27%	0.009
U. m. of *Enterococcaceae* family	0.01%	0.03%	0.46%	192.58%	0.11	1623.84%	0.008	4943.66%	0.02
Enterococcus	0.27%	1.95%	16.86%	629.45%	0.03	765.98%	0.0008	6216.91%	0.0005
Staphylococcus	0.01%	0.02%	0.64%	184.36%	0.39	2867.41%	0.09	8338.06%	0.2
Klebsiella	0.12%	0.19%	14.31%	54.25%	0.48	7429.84%	0.008	11514.90%	0.002
Corynebacterium	0.00%	0.00%	0.25%	-12.20%	0.2	21185.17%	0.3	18588.67%	0.72
Hafnia	0.00%	0.00%	0.19%	-83.81%	0.19	129552.00%	0.31	20894.04%	0.28
Citrobacter	0.01%	0.01%	4.39%	169.45%	0.67	30311.42%	0.35	81844.29%	0.25
Proteus	0.00%	0.00%	0.99%	114.82%	0.27	96585.45%	0.39	207601.83%	0.33

* Age- and sex-adjusted

° Taxa in bold show decrease lower than 50% in CDI respect to AB- subjects with p-value <0.005

✦Taxa underlined show decrease lower than 50% or increase higher than 100% in AB+ and CDI respect to AB- subjects

U.m. = Unclassified members.

Table S3. Shotgun metagenomics datasets filtering table.

Sample	Number of reads	Final read number
7AB+	454948	167574
17AB+	817736	660670
18AB+	858782	508302
27AB+	533960	345756
28AB+	470413	372703
4CDI	594428	496810
6CDI	217508	132353
9CDI	608073	527978
14CDI	685437	191471
19CDI	500078	391433
4AB-	142807	121962
12AB-	225933	175843
18AB-	914260	209705
19AB-	215631	164462
22AB-	667180	571514

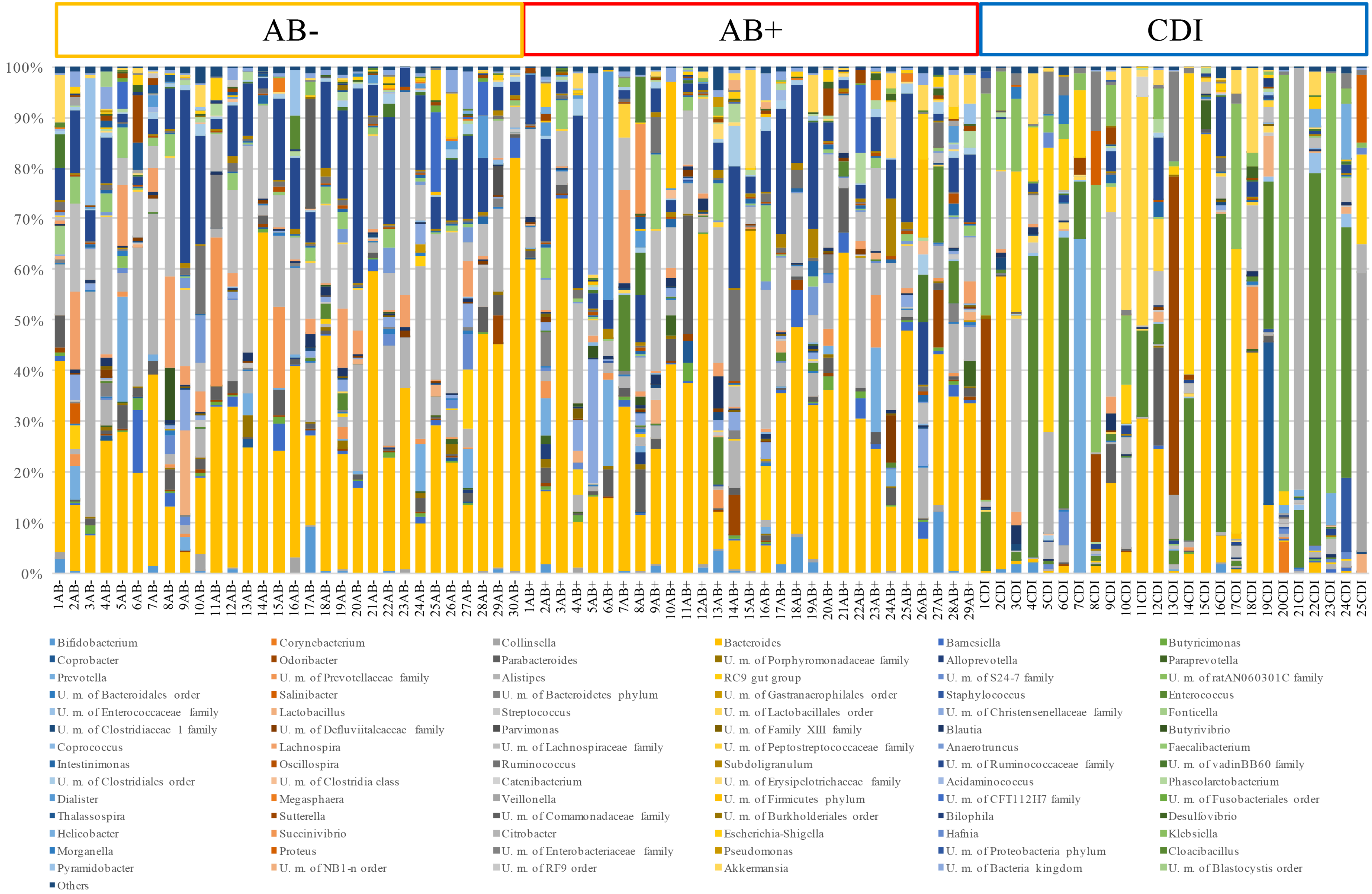

* U. m. of = Unclassified member of

Figure S1

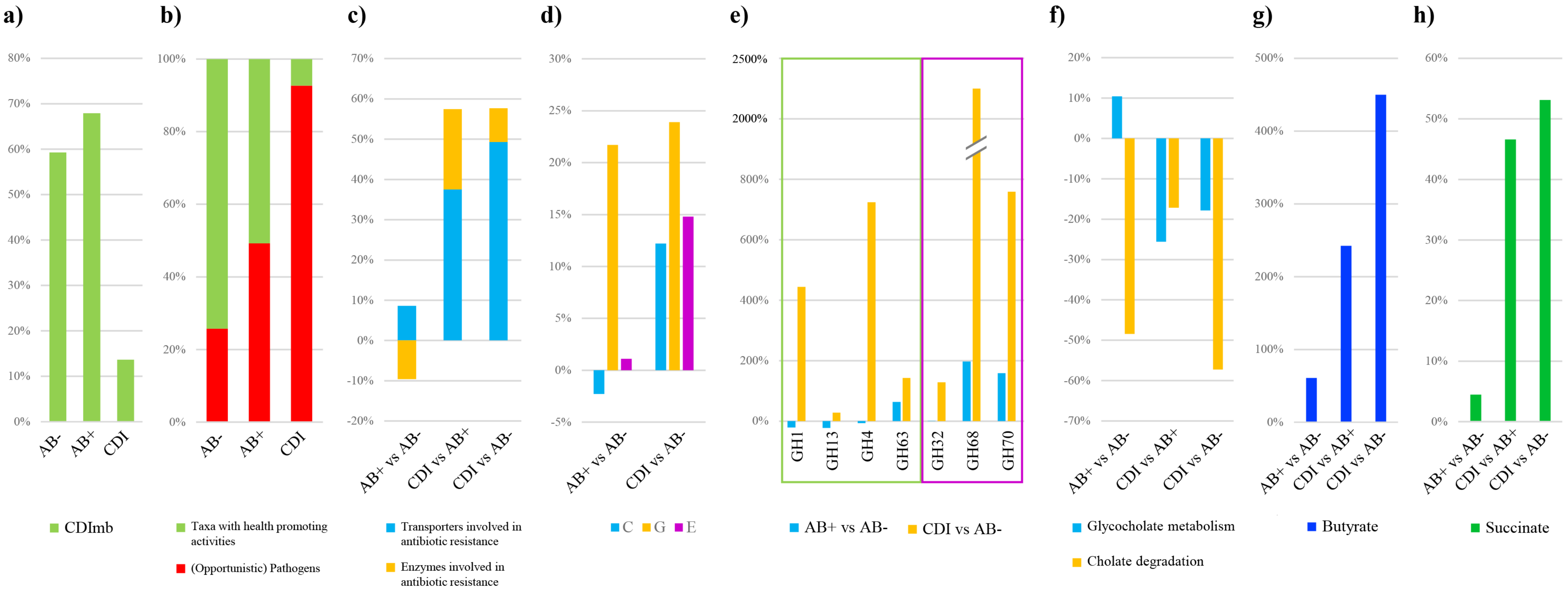

a)
b)
c)
d)
e)
f)
g)
h)
AB-
AB+
CDI
CDImb
Taxa with health promoting activities
(Opportunistic) Pathogens
AB+ vs AB-
CDI vs AB+
CDI vs AB-
Transporters involved in antibiotic resistance
Enzymes involved in antibiotic resistance
C
G
E
GH1
GH13
GH4
GH63
GH32
GH68
GH70
AB+ vs AB-
CDI vs AB-
Glycocholate metabolism
Cholate degradation
Butyrate
Succinate

Figure S2

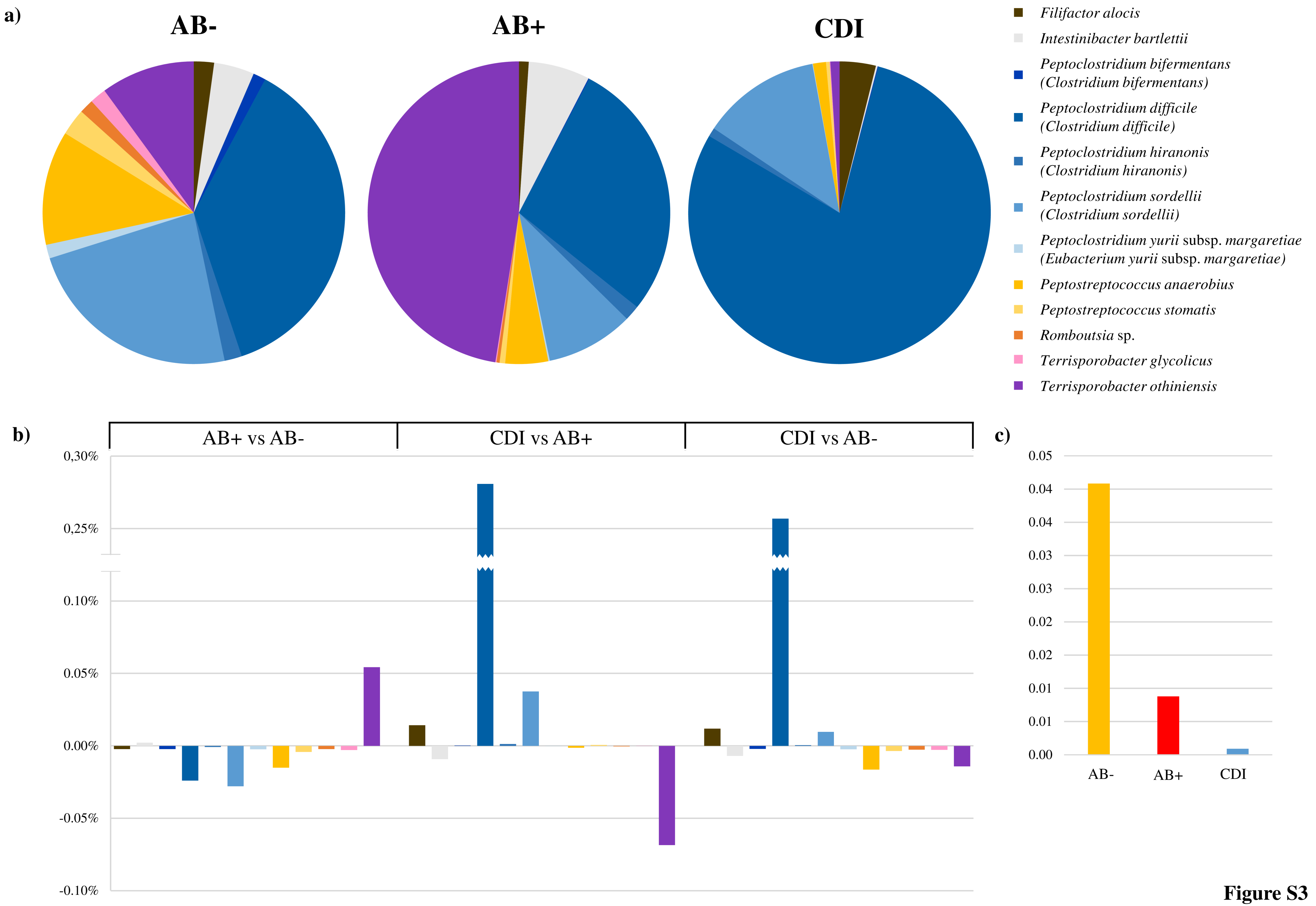
a)
AB-
AB+
CDI
Filifactor alocis
Intestinibacter bartlettii
Peptoclostridium bifermentans (Clostridium bifermentans)
Peptoclostridium difficile (Clostridium difficile)
Peptoclostridium hiranonis (Clostridium hiranonis)
Peptoclostridium sordellii (Clostridium sordellii)
Peptoclostridium yurii subsp. margaretiae (Eubacterium yurii subsp. margaretiae)
Peptostreptococcus anaerobius
Peptostreptococcus stomatis
Romboutsia sp.
Terrisporobacter glycolicus
Terrisporobacter othiniensis
b)
AB+ vs AB-
CDI vs AB+
CDI vs AB-
0,30%
0,25%
0.10%
0.05%
0.00%
-0.05%
-0.10%
c)
0.05
0.04
0.04
0.03
0.03
0.02
0.02
0.01
0.01
0.00
AB-
AB+
CDI

Figure S3
